# Improved tendon healing by a combination of Tanshinone IIA and miR-29b inhibitor treatment through preventing tendon adhesion and enhancing tendon strength

**DOI:** 10.7150/ijms.44138

**Published:** 2020-04-27

**Authors:** Haiying Zhou, Shuai Jiang, Pengfei Li, Hui Shen, Hu Yang, Shengquan Xu, Chenyi Ye, Mingjian Chen, Hui Lu

**Affiliations:** 1 Department of Orthopaedics, The First Affiliated Hospital, College of Medicine, Zhejiang University. 79 Qingchun Road, Hangzhou, Zhejiang Province, P.R.China, 310003; 2 Department of Plastic and Aesthetic Center, The First Affiliated Hospital, College of Medicine, Zhejiang University. 79 Qingchun Road, Hangzhou, Zhejiang Province, P.R.China, 310003; 3 Orthopedics Research Institute, Zhejiang University, Hangzhou 310000, China

**Keywords:** miR-29b, Tanshinone IIA, tendon adhesion, tendon repair

## Abstract

**Background**: Despite significant advances in the materials and methods development used in surgical repair and postoperative rehabilitation, the adhesion formation remains the most common clinical problem in tendon injuries. Therefore, the development of novel therapies is necessary for targeting at preventing tendon adhesion formation and improving tendon strength.

**Methods**: We used rat fibroblasts for in vitro experiments to determine the optimal concentration of TSA in rats, and then set up negative control group, TSA intervention group, mir-29b interference adenovirus intervention group and TSA and mir-29b interference adenovirus co-intervention group. By comparing cell proliferation and protein expression in different group, we verified the effect and mechanism of drugs on fibroblast function. At the same time, the Sprague-Dawley rat Achilles tendon model* in vivo* was established in this study, which was divided into sham operation group and operation group. Afterwards in the operation group, mir-29b inhibitor and placebo were injected every 3 days respectively. Then the injection inhibitor group was divided into 5 groups which mean TSA was injected into the marked area at 0, 6, 24 and 72 hours after operation for 1 week, finally all of the rats were died at 3 weeks after operation. Through the observation of general properties, histological observation of Achilles tendon injury, biomechanical test and cell and protein expression in rats' tendon cell, the effect of drugs on tendon adhesion formation was analyzed.

**Results**: We demonstrated that the combination of miR-29b inhibitor and tanshinone IIA(TSA) could prevent tendon adhesion and also enhance tendon strength. Mechanically, the miR-29b inhibitor could activate the TGF-β/Smad3 pathway to trigger endogenous pathways and induce a high proliferation of fibroblast. Subsequently, we also found adding TSA after 6 hours of miR-29b treatment gave less cell cytotoxicity in our rat model with better outcome of less tendon adhesion and enhanced strength.

**Conclusion**: We conclude that the use of miR-29b inhibitor at the end of the tendon break could initiate endogenous repair mechanism and subsequently use of TSA should be able to inhibit the exogenous repair mechanism. Therefore, the combination of both treatments could prevent tendon adhesion and ensure tendon strength. Our findings suggested that this approach would be a feasible approach for tendon repair.

## Background

Tendon is a highly organized tissue that connects muscle to bone and facilitates joint movements. Due to overuse or aging-related degeneration, tendon injuries become one of the most common clinical problems 1, 2, while tendon healing is a complex process which mainly involving both endogenous and exogenous mechanisms 3. Despite advances in the materials and methods used in surgical repair and postoperative rehabilitation 2, tendon adhesion is considered as the significant problem during the processing of wound healing after surgery 4. Studies have been shown that inflammation at the early stage of healing is the leading cause of soft tissue adhesion 3, 5, 6. Also, tendon healing usually cannot fully restore normal tendon strength and results in significant weakness 7. There are many biological materials have been evaluated to prevent tendon adhesion, such as amniotic membrane and seprafilm. However, the common problem is that those materials induce inflammation and have very poor cellularity 8, 9. Also, several non-steroidal anti-inflammatory drugs such as ibuprofen have been evaluated from tendon injury treatment 10, 11. However, due to their complexity and restriction of tendon adhesion, reliable therapies are still missing. Therefore, it is necessary to develop new practical approaches for tendon healing.

Tanshinone IIA (TSA) is a member of the most abundant lipophilic components extracted from *Salvia miltiorrhiza* 12. Studies have been shown that TSA has anti-inflammatory activity and has been widely used for various diseases 13, 14. In our recent studies and others demonstrated that TSA could effectively prevent tendon adhesion through interaction with TGF-β/Smad signaling pathway 12, 15. However, studies have not investigated the treatment effects on tendon strength. Many studies have shown that microRNA is emerging as vital homeostatic regulators of tissue repair 16. The miRNA-29 (miR-29) plays an important role in the regulation of key process of fibrosis and scar formation 17. In our previous study, we also found that TSA treatment enhanced the expression level of miR-29b 12. Indeed, the miR-29b has been shown to play an important role in many different diseases such as diabetes, cancer, and heart. In particular, previous studies found that miR-29b is a downstream inhibitor of TGF-β/Smad3-mediated fibrosis and treatment of miR-29b had therapeutic potential for disease associated with fibrosis 15, 18-21. In summary, previous studies have shown that the miR-29b can prevent tendon adhesion through downregulation of the TGF-β1 and Smad3 expression and TSA can inhibit TGF-β1 to prevent tendon adhesion as well. As we just mentioned that tendon healing involved both endogenous and exogenous mechanisms.

We hypothesized that the use of miR-29b inhibitor at the end of tendon break could initiate endogenous repair mechanism and subsequently use of TSA should be able to inhibit exogenous repair mechanisms, therefore prevent tendon adhesion while ensuring tendon strength. By using Sprague-Dawley rat model, we demonstrated that the combination of miR-29b inhibitor and TSA treatment not only prevent the tendon tissue adhesion but also increase tendon strength.

## Materials and Methods

### Establishment of rat tendon model

All animal experiments were approved by animal committee for ethics of laboratory animal research center, First Affiliated Hospital, College of Medicine, Zhejiang University and the animal experiment procedures were approved by the Institutional Animal Care and Use Committee, First Affiliated Hospital, College of Medicine, Zhejiang University. The rat model of adhesion of tendon was eatablished as in our previous studies 12, 15. Briefly, rats were anesthetized using halothane (50 mg/kg weight). Under general anesthesia, a longitudinal incision was made from the heel to hind paw under a control tourniquet. Achilles tendon was half partial lacerations involved approximately 50% of tendon fibers. The tendon was repaired by a modified Kessler's technique with 5-0 Ti-Cron coated braided polyester sutures (COVIDIEN, USA). Then the tendon sheath was closed with the same sutures. The skin was closed with 5-0 Surgipro monofilament polypropylene (COVIDIEN, USA). After surgery, rats were allowed to perform free active motion and regular weight-bearing.

### Isolation of fibroblasts

Scar tissues of repaired tendon sites from Male Sprague-Dawley rat hind paws were dissected out and placed into laminar flow cabinet and minced into 5mm tissue pieces and seeded into a petri dish. Fibroblasts can be isolated in D10 medium containing 10% fetal bovine serum, 1% Penicillin/streptomycin, and 200U/ml collagenase in DMEM medium. The suspension was filtered to get fibroblast cells and then cultured with D10 at 37 °C.

### MTT assay

The cytotoxicity of Tanshinone IIA was determined using the MTT assay. Fibroblast cells were resuspended into 5 x10^4^ cell/ml and put 100μl of the cell suspension to the 96-well plates. The cells were cultured overnight, and TSA at various concentrations was added to the culture ranging from 0, 0.001, 0.01, 0.1, 1,10 and 20 μM, respectively. Then the cells were further incubated for 24 hours. After incubation, the supernatant was removed, 100 μl of DMSO was added to each well and incubated for 10 min. A microplate reader was used to measure at 570 nm. All experiments were performed in triplicate. The viability was calculated using the formula: % viability = value of treated cells/ value of control cells x 100%.

### CCK-8 assay

Rat fibroblasts in logarithmic growth phase were inoculated in 96-well plate, with the amount of 100 μl per well and the cell suspension density of 5×10^4^/ml. The cells were treated with DMSO, scramble siRNA control, miR-29b shRNA, TSA, scramble siRNA control and TSA, miR-29b shRNA and TSA, respectively. μl of CCK-8 was added to each well (Beyotime). After 4 h of incubation, the OD450 value was measured by spectrophotometer. Viability (%) was calculated based on the optical density (OD) values, as follows: (OD of TSA treated sample - blank) / (OD of control sample - blank) ×100.

### TUNEL assay

The tissue from rat model under different treatment conditions was fixed with 4% paraformaldehyde in PBS, permeabilized by 0.1% Triton X-100 and followed by treating with TUNEL reagent (Beyotime Institute of Biotechnology, Haimen, China) and then, incubated for 30 min at room temperature. Images were captured using a BX53 microscope (Olympus Corporation, Tokyo, Japan). The cell nuclei were stained with Alexa Fluor 647 and DAPI. The percentage of TUNEL-positive cells in the total cell population was calculated.

### Quantitative Real-time Polymerase Chain Reaction

Total RNAs was extracted from primary fibroblasts or rat tissue using TRIzol (Invitrogen) according to the manufacturer's instructions. Then, 1-2μg of total RNA was reverse transcribed using a HiScript Reverse Transcriptase (Vazyme), and then the cDNA was quantified using the SYBR Green I with Taq Plus DNA Polymerase. After a circle reaction, the threshold cycle (Ct) was determined, and the relative expression levels of TGF-β1, Col1, Col3, Cyclin D, p21, Smad3 were calculated based on the Ct values normalized to GAPDH in each sample. The relative expression of miR-29b was detected using a Bulge-Loop miRNA qRT-PCR kit (RiboBio) with U6 as the internal control. The sequences of the primers in this study were shown in Table S1.

### Western Blot

For western blot analysis, total proteins were extracted from tissues or primary fibroblasts. The BCA(bicinchoninic acid) method was used to qualify the amount of protein with BSA standard. A total of 40 μg of protein was fractionated on a 12% SDS-polyacrylamide gel and transferred to a polyvinylidene fluoride membrane (PVDF). The membrane was then blocked with 5% milk in a Tris-buffered saline solution (TBST) for 2 hours at room temperature. The primary antibodies, TGF-β1 (1:1000; Abcam), p21 (1:1000; Abcam), Smad3(1:1000, Abcam), pSmad3 (1:3000, Cell Signaling) and GAPDH (1:1000; Xianzhi Bio, Hangzhou, China) were used and incubated overnight at 4°C, followed by incubation with horseradish peroxidase (HRP)-conjugated secondary antibody (1:50,000, Boshida, Wuhan, China). The proteins were visualized, and band density was quantified by using BandScan software.

### Transfection Procedure

The transfection procedure was performed using Lipofectamine 3000 according to the manufacturer's instructions. Briefly, the cells were washed with serum-free and antibiotics-free DMEM and then replaced with serum-free and antibiotics-free DMEM for 6 hours prior to transfection. The miRNA mimics or miRNA inhibitor (RiboBio, Guangzhou, China) and Lipofectamine 3000 (Invitrogen) were separately mixed using Opti-MEM medium (Gibco, China) for 5 minutes, then two components were subsequently mixed and further incubated at RT for 20 minutes. The DNA-lipid mix was added to the cells and further cultured for 6 hours. Fresh medium was replaced after 6 hours of incubations with transfection reagents.

### Annexin V assay

To determine cell apoptosis in primary fibroblasts cells under different treatment conditions, we performed Annexin V staining. The samples were stained with 5ul of Annexin V (APC) for apoptotic cells and 5ul of 7-AAD for dead cells. The cells were washed and analyzed by flow cytometry using cytoFlex (Beckman coulter).

### Cell Cycle Analysis

The cell cycle was analyzed by flow cytometry. Briefly, primary fibroblast cells were treated under different conditions was used for analysis. After treatment, cells were collected and washed twice with ice-cold 1 × PBS, suspended in 700μl of pre-cooled 80% alcohol, and fixed the cells at 4°C for 4 hours. The cells were then stained with the Cell cycle kit (Keygen, Nanjing, China). The cells were washed and analyzed by flow cytometry using cytoFlex (Beckman coulter).

### Histological assessment and Masson trichrome staining

The rats were sacrificed 3 weeks after surgery, and the harvested specimens were immediately fixed in 4% paraformaldehyde, dehydrated in ethanol and then embedded in paraffin blocks. Histological sections (4μm) were prepared for hematoxylin and eosin staining (H&E). The quantity and ratio of fibroblast-like cells within the repaired tissue were assessed. Also, Masson trichrome staining was performed according to standard procedures to examine the general appearance of collagen deposition and collagen fibers.

### Rat animal model experiment

We selected 8-week-old male SD rats for the experiment, 5 rats in each small group. Firstly, we divided rats into sham operation group (5 rats) and operation group (25 rats). After the Achilles tendon injury model was established in the operation group, there were two different treatment one (20 rats) was miR-29b inhibitor and the other (5 rats) was negative control, things were injected every three days. Then in the inhibitor group, there were four different injection time which divided them in four small group, and the drugs of TSA were injected at 0h, 6h, 24h and 72h, respectively through the marker area for 1 week. Three weeks later, the rats were sacrificed, Achilles tendon defects of 6 mm in length were generated by full-thickness cut-outs in SD rats. The biomechanical test was performed using a universal tensile test machine at a rate of 5mm/min until failure, and the histological and protein expression changes were detected by the above methods.

### Statistical analysis

Data were expressed as mean ± Standard error. Statistical significance was determined by two-way ANOVA with Tukey multiple comparisons test and default setting. A p-value less than 0.05 were considered significantly different. All the graphs statistics were analyzed by GraphPad prism version 8.2.1.441. The figures were prepared by adobe illustrator 2019.

## Results

### TSA and miR-29b inhibitor demonstrated opposite effects on tendon adhesion

**Figure 1:**
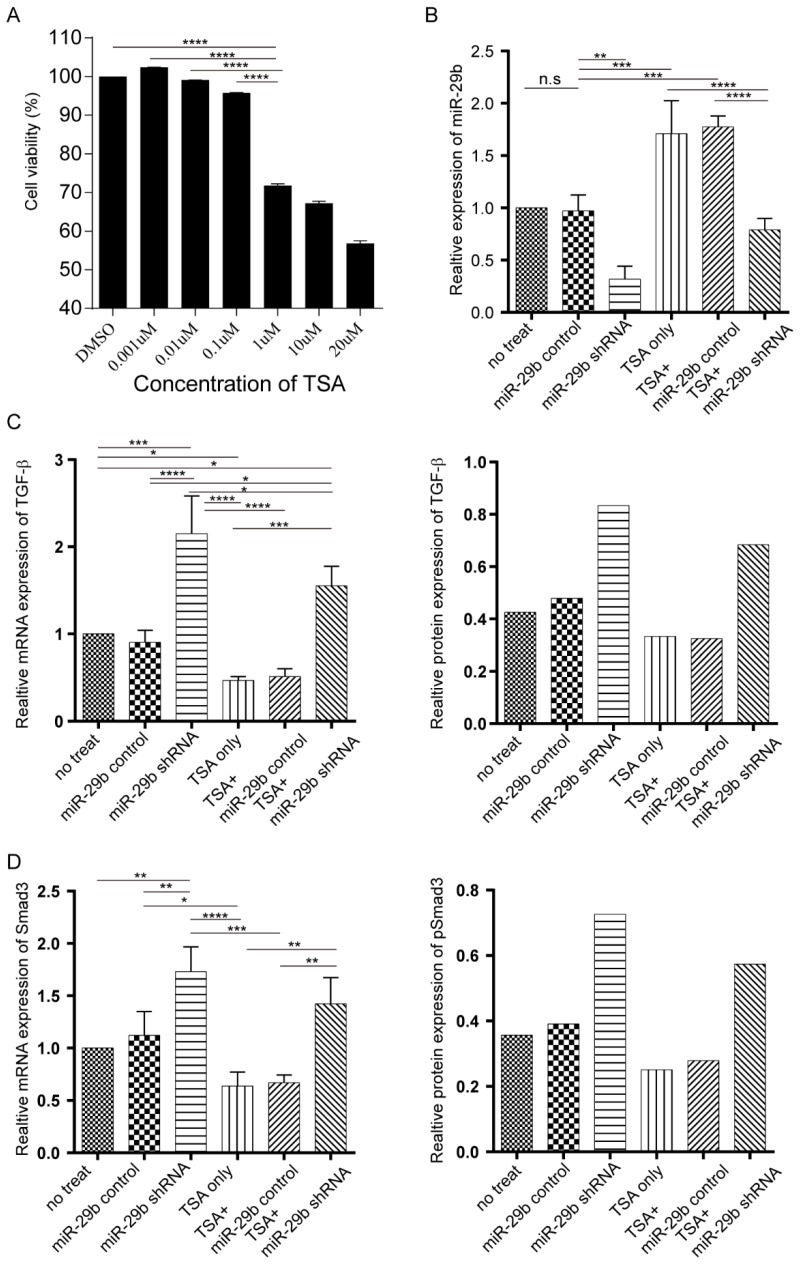
The dynamic changes of miR-29b, TGF-β, and Smad under miR-29b inhibitor and TSA treatment. A: the cytotoxicity of TSA was determined by MTT assay. B: miR-29 expression was measured by qPCR. C: both mRNA and protein expression level of TGF-β were measured under different conditions. D: the Smad mRNA expression was measured by qPCR (n=3) and protein expression level (n=1) were measured by western blotting under different conditions., p-value *<0.05, **<0.01, ***<0.005, ****<0.001

In our recent study using Sprague-Dawley rat model, we demonstrated that TSA treatment could prevent tendon adhesion but fail to enhance the tendon strength 12, 15. To test whether using a combination of TSA and miR-29b inhibitor could result in better treatment of tendon adhesion and enhance tendon strength through modulating endogenous and exogenous repair pathways, we first tested on primary fibroblast cells *in vitro* isolated from the rat. The MTT results indicated that TSA at 1μM significantly reduced cell viability after 24 h of treatment. Hence, we use 0.1μM TSA in this study (Figure 1A). We next investigated the effects of both TSA and miR-29b inhibitor treatment using primary rat fibroblast cells. The shRNA silencing of miR-29b clearly showed downregulation of miR-29b in fibroblast cells, and treatment of TSA significantly enhanced the expression of the miR-29b, which was consistent with our previous studies 12, 15. Strikingly, simultaneous treatment of cells with TSA and miR-29b shRNA counteract the effects of the treatment showing that there are no significant changes of miR-29b in double-treated samples (Figure 1B). Our previous studies showed treatment with TSA alone could prevent tendon adhesion through TGF-β/Smad signaling pathway, therefore, we investigated the dynamic changes of TGF-β and Smad expression in both mRNA and protein level under different treatment conditions. Consistent with our previous study, we found that TSA treatment decreased the expression of both TGF-β and Samd3 level. In contrast, the miR-29 inhibitor significantly upregulated the expression of both TGF-β and Smad3 (Figure 1C-D and Figure S1A). Strikingly, when the cells treated with both TSA and miR-29 inhibitor at the same time, we observed that the expression level of TGF-β and Samd3 were significantly higher than TSA treated only, but significantly attenuated compared to the sample treated with miR-29b inhibitor. Our findings confirmed that both TSA and miR-29b inhibitor target the same pathway implying that the combination could trigger endogenous pathways and manipulate late stage of targeting at exogenous pathways.

### Effects of TSA and miR-29b on cell proliferation and cell cycles

**Figure 2:**
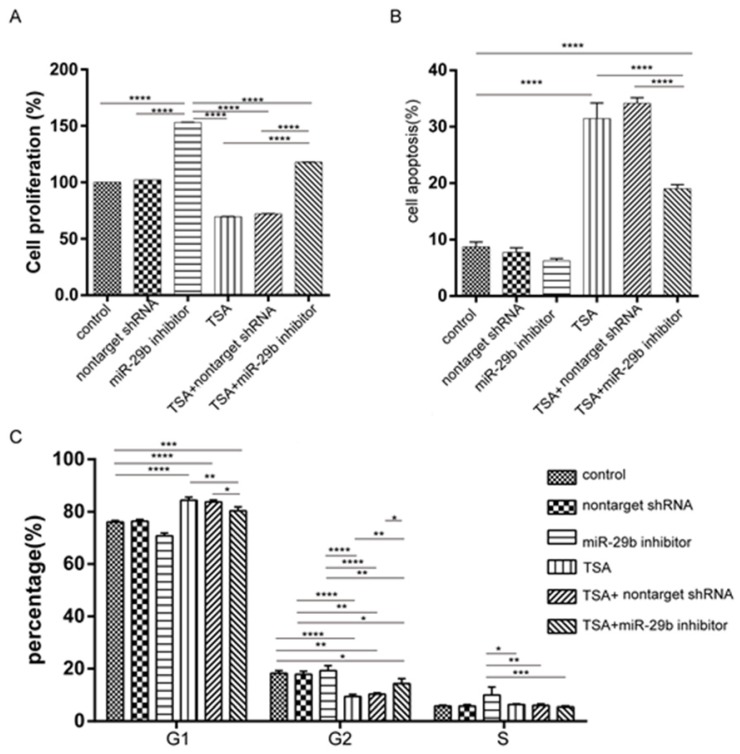
Effects of cell proliferation, apoptosis, and cell cycles under miR-29b inhibitor and TSA treatment. A: cell proliferation was performed by CCK8 kit. B: Annexin V and PI staining were performed by FACS. The summarized results were shown here. C: Cell cycle analysis of primary isolated cell treated under different conditions. Cells were cultured with different concentrations of TSA for 24 h and then stained with propidium iodide. The DNA content was analyzed by flow cytometry. n=3, p-value *<0.05, **<0.01, ***<0.005, ****<0.001

To test the capability using the combination of TSA and miR-29b inhibitor for treatment, we further investigated the cytotoxicity effects and cell proliferation in primary cell models. The CCK-8 assay demonstrated that cells treated with miR-29b inhibitors significantly increased cell proliferation (Figure 2A), while TSA treated cells significantly decreased cell proliferation compared with no treated cells which are consistent in our previous study (Figure 2A). Interestingly, when the cells treated with both TSA and miR-29b inhibitor, we found that cell proliferation ability was significantly decreased when compared with the miR-29b inhibitor only and higher than the TSA treated cells. The same trends were observed in cell apoptosis analysis which mean opposite result of apoptosis compared with cell proliferation in three treatment group, these further suggesting the antagonistic effects of TSA and miR-29b inhibitor. It has been described that the dynamics of cell growth in different treatment conditions may result from cell cycles, cell death, or a combination of these two processes. Thus, we next investigated the cell cycle distribution and cell apoptosis under different conditions by fluorescence-activated cell sorting (FACS) analysis, which gives a measure of DNA content, to explore the related mechanism of the growth dynamic for each different treatment.

As shown in Figure 2C, the TSA treated cells exhibited a higher proportion of cells in the G1 phase, while the TSA induced higher cell apoptosis as compared with control cells. Besides, we observed a concomitant decrease in the proportion of cells in the G2 and S phase in the TSA treated groups compared with the miR-29b inhibitor-treated ones Figure S1B and 1C). At the same time, when the cells were treated with both TSA and miR-29b inhibitor, we also observed that cell apoptosis was significantly reduced and had slightly higher proportion of cells in the G1 phase compared to TSA treated only, but opposite to miR-29b inhibitor treated only. In sum, the results suggested that miR-29b was able to increase cell proliferation and TSA treatment could reduce those effects.

### The combination of TSA and miR-29b inhibitor treatment increase cell proliferation

**Figure 3:**
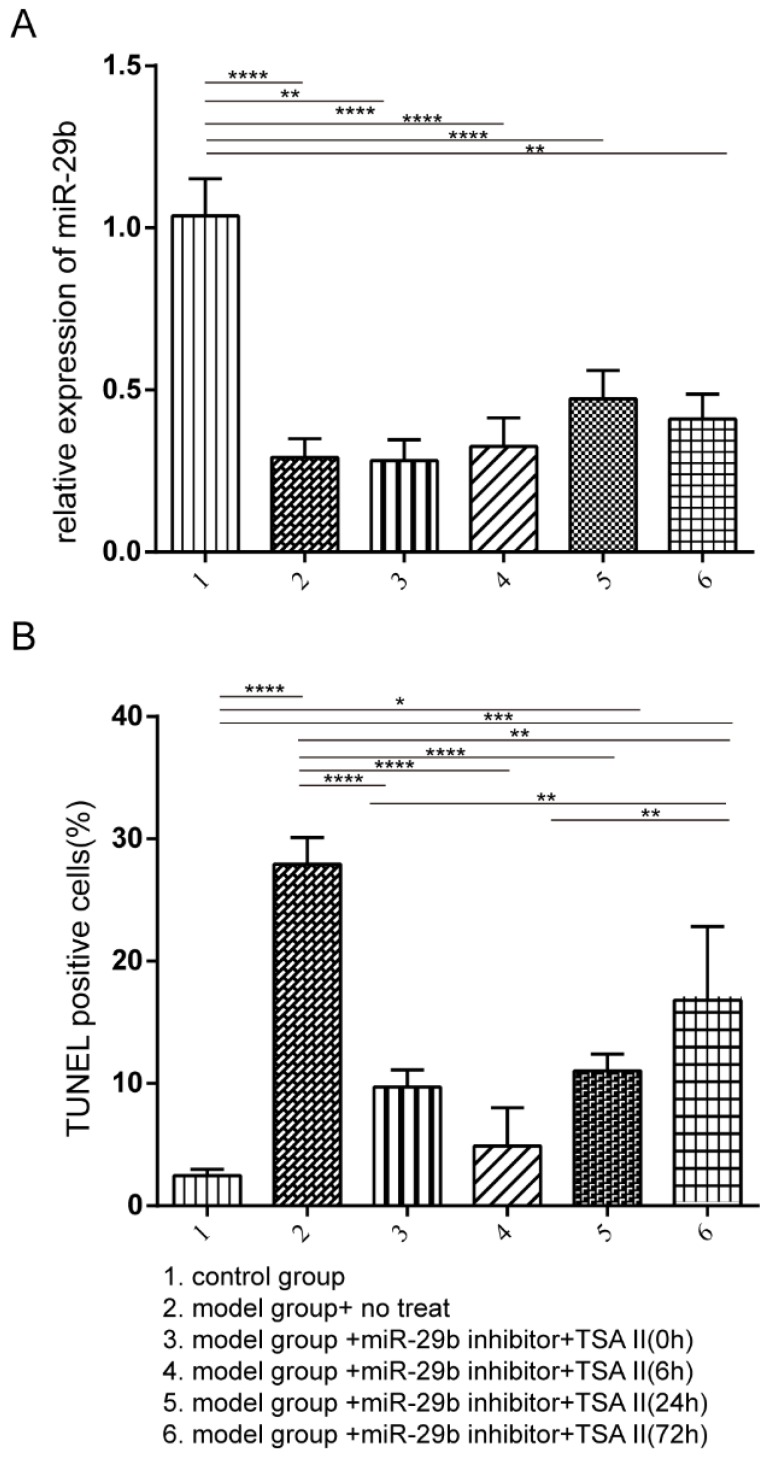
The rat model was established and treated with the different condition as described from Group 1 to 6. The rat was then sacrificed after three-week after treatment. A: the miR-29b expression was quantified by qPCR, and the result was normalized to the control group. B: TUNEL assay was performed to investigate the frequency of apoptotic cells. n=3, p-value *<0.05, **<0.01, ***<0.005, ****<0.001

Our *in vitro* primary cell results demonstrated that treatment of TSA could reduce the expression of TGF-β and Smad3, and alter the cell proliferation and apoptosis, in contrast, the miR-29b inhibitor treatment showed the opposite effects. However, when treated cells by using the combination of TSA and miR-29b inhibitor, we clearly observed antagonistic effects suggesting that we could first treat cells with the miR-29b inhibitor to enhance endogenous repair pathway and then followed with the TSA treatment to reduce the tendon adhesion formation. The male Sprague-Dawley rat animal model was used to test the hypothesis, the rat was treated with or without miR-29b inhibitor to induce endogenous repair, and then, the TSA was added at different time-points as indicated in Figure 3. We first investigated the efficacy of miR-29b inhibitor in our rat model, and the results indicated that all inhibitor treatments significantly suppressed the expression of miR-29b RNA compared with untreated control as shown in Figure 3A. Besides, we investigated the cytotoxicity of the TSA and miR-29b inhibitor in different treatment conditions. Our results demonstrated that the combination of TSA and miR-29b inhibitor treatments significantly decreased the number of apoptotic cells. Strikingly, we observed that the rat treated with miR-29b inhibitor first and followed by TSA after 6 hours showed slightly lower level of apoptotic cells compared with other treatment while closed to control rat groups suggesting that different treatment time-point could result in distinct treatment effects.

### Both TSA and miR-29b inhibitor regulated TGF-β/Smad3 pathway

**Figure 4:**
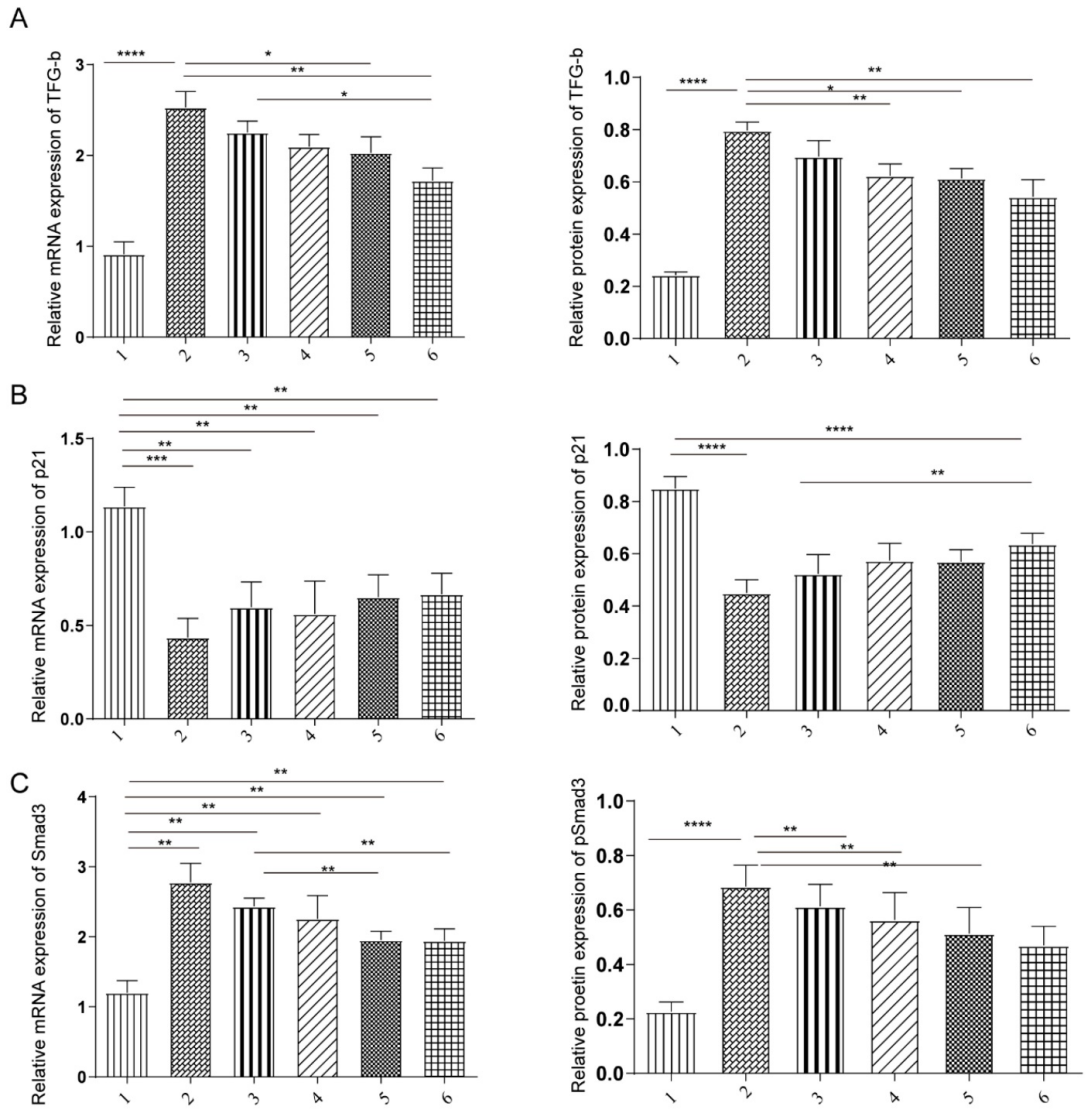
The effects of under miR-29b inhibitor and TSA treatment on TGF-β, p21, and Smad3 expression. A: the TGF-β mRNA expression was measured by qPCR and protein expression level were measured, n=3. B: the p21 mRNA expression was measured by qPCR and protein expression level were measured, n=3. C: the Samd3 mRNA expression was measured by qPCR and protein expression level were measured, n=3, p-value *<0.05, **<0.01, ***<0.005, ****<0.001

Our previous study and *in vitro* data suggested that both TSA and miR-29b inhibitor treatment were through TGF-β1/Smad3 pathway. We next investigated that molecular mechanism of TSA and miR-29b inhibitors in the rat model. The expression level of TGF-β, p21 and Smad3 were measured at mRNA and protein levels under different treatment conditions. We observed that the expression was consistent in both mRNA and protein level, showing that treatment decreased the level of TFG-β and Smad3 level (Figure 4A-C). However, the TGF-β and Smad3 level still higher than healthy control, suggesting that the treatment might not heal the tendon completely which needed to be further investigated in more details.

### Effects of Treatment of TSA and miR-29b inhibitor on collagen expression and fibroblast proliferation

**Figure 5:**
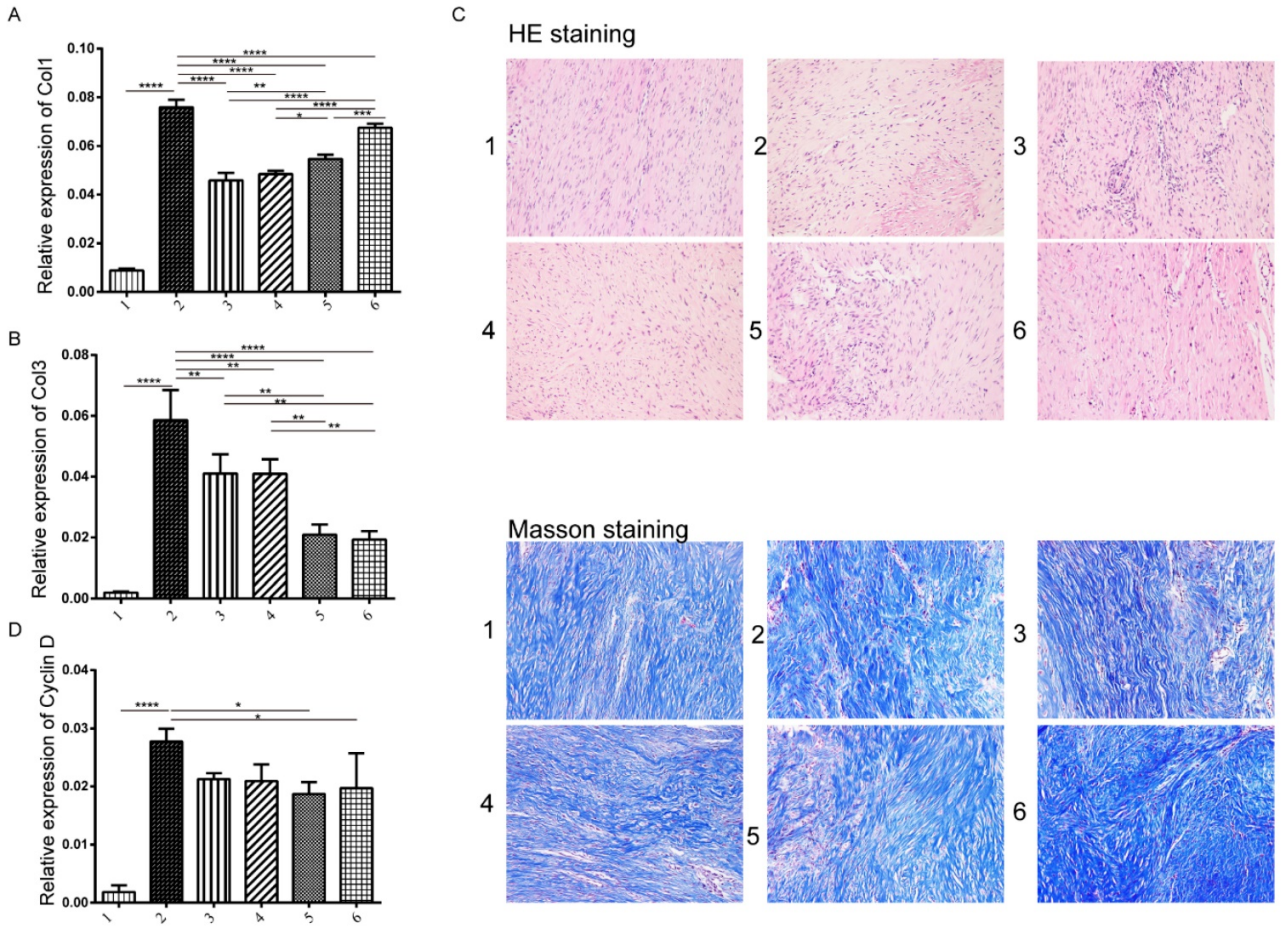
Analysis of tendon tissue on collagen expression and histology changes. The collagen I expression level was measured by immunohistochemistry in each group at three weeks. A: the production of collagen I in different groups B: the production of collagen III, C: Hematoxylin & Eosin (H&E) staining (upper panel) and Masson staining (lower panel) on tendon tissue of rats in various groups (×200). D: the expression of cyclin D: histological evaluation of tendon tissue at weeks 3 in each group. n=3, p-value *<0.05, **<0.01, ***<0.005, ****<0.001

Previous studies have shown that fibroblast proliferation and collagen I and III (Col I/III) expression are the two major factors contributing to the formation of tendon adhesions (22, 23. Therefore, we next tested the expression level of Col I/Col III and Cyclin D to look at collagen expression and fibroblast proliferation Figure S3. We observed that Col I and Col III showed opposite trends which showed that the protein levels of Col I increased when delayed the addition of TSA treatment, while the cyclin D showed the significant increase compared to control group but no differences among disease model groups (Figure 5A-C). We next evaluated the histological repaired tissue by HE and Masson staining Figure S2. Among all groups, we observed that fibroblasts and collagenous tissue proliferated at the repaired site compared with the untreated control, and furthermore, the number of collagen fiber and fibroblast increased and well arranged in the group treated miR-29b inhibitor and TSA (Figure 5D).

### Effects of Treatment of TSA and miR-29b inhibitor on tendon strength and adhesion

**Figure 6:**
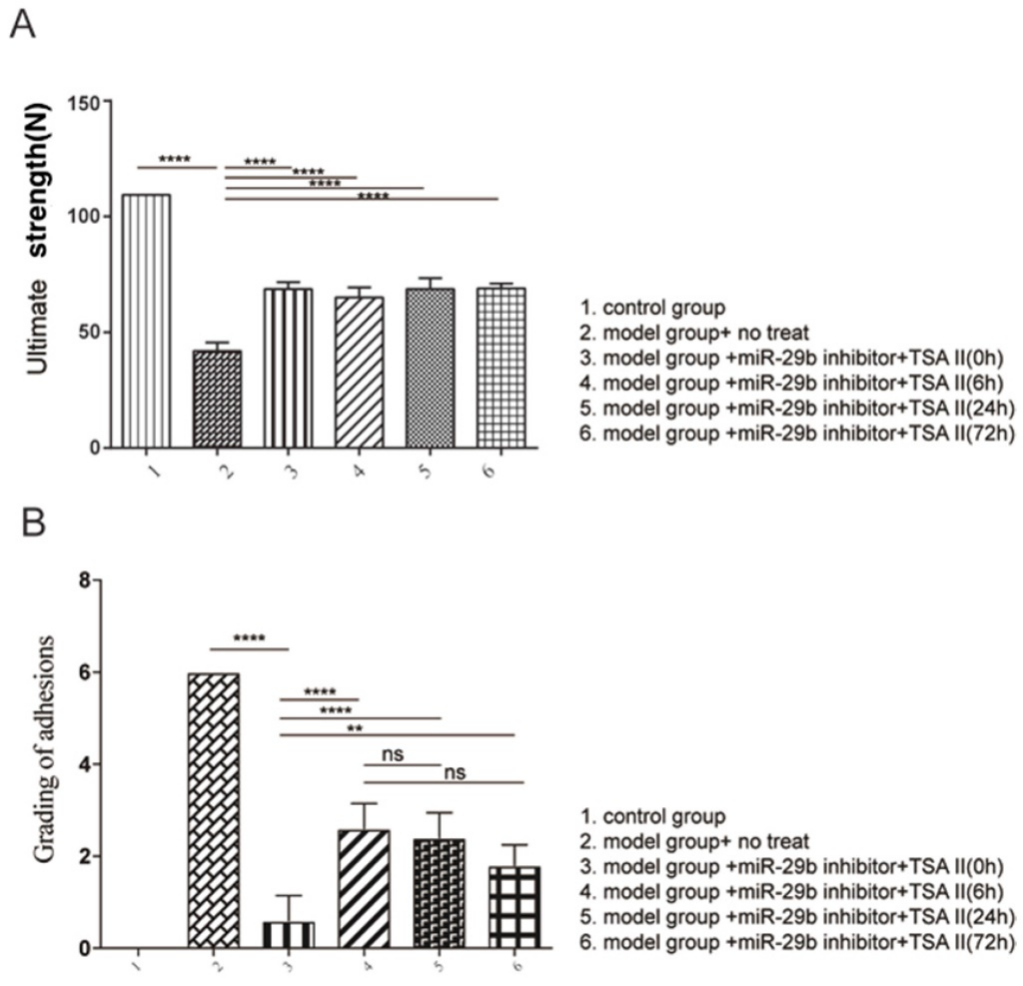
analysis of tendon strength. A: Biomechanical analysis of maximum load was analyzed. B: evaluation of peritendinous adhesions. n=3, p-value *<0.05, **<0.01, ***<0.005, ****<0.001

We next evaluated the standard degree of adhesion according to the previous study 12. The results indicated that the combination of treatment reduced tendon adhesion (Figure 6A). Moreover, the tendon strength under different treatment was evaluated in the current study (Figure 6B). Our results indicated that all treatment conditions resulted in higher maximum loads than disease model, suggesting that early induce endogenous repair mechanism could increase the tendon strength; and that there is no adverse effect on tendon adhesion.

## Discussion

In this study, we investigated the treatment effects on tendon adhesion and healing using *in vitro* and *in vivo* model treated with the combination of miR-29b inhibitor and TSA, we showed that using miR-29b inhibitor could increase cell proliferation and also active TGF-β/Smad3 pathway to enhance the endogenous healing processes and the followed TSA treatment could negatively modulate the TGF-β/Smad3 pathway to prevent tendon adhesion formation.

Tendon healing is a complex clinical process involving simultaneous endogenous and exogenous repair. Tendon adhesion is regarded as one of the most significant problems of wound healing after surgery 12, 14, 24. Tendon healing typically included exogenous and endogenous healing mechanisms. Endogenous healing mainly involves in inducing tenocytes proliferation and exogenous healing occurs through the chemotaxis of specialized fibroblasts and inflammatory cells from the periphery, blood vessels, and circulation into the defect from the ends of the tendon sheath. The tendon strength is heavily depending on endogenous healing mechanisms, while the exogenous mechanism is the main problem induce tendon adhesion 25, 26. In general, the exogenous pathway is induced earlier than endogenous pathway because endogenous pathway happened as early as 24 hours 27. Previous studies have been mainly focused on how to modulate exogenous pathway while haven't paid much attention to endogenous pathway^1^. Therefore, in our current study, we used both miR-29b inhibitor and TSA combination aim to address this issue.

TSA is an effective component of *Salvia miltiorrhiza*, a plant of Salvia in Labiatae, which has antibacterial, anti-inflammatory, vasodilative and anti platelet aggregation functions. It has been proved to be closely related to tissue fibrosis 28, 29. In the Hepatic fibrosis model of mice that was induced by intraperitoneal injection of thioacetamide (TAA), the treatment of TSA lead to the lower expression of alpha-SMA, collagen I, TGF-beta1, Smad3 and IGFBP7 in liver, which implicated that TSA could improve liver function and inhibit the fibrosis through blocking TGF-β1/Smad3 signaling pathway^30^. At the same time, researchers 31 found that TSA could play as an antioxidant in the high glucose cultured cardiac fibroblasts, where it could inhibit the TGF-β1/Smad to reduce the high glucose-mediated collagen synthesis, and ultimately inhibit myocardial fibrosis. Wang et al. 32 confirmed that TSA can reduce renal fibrosis and inflammation in rats after 5/6 nephrectomy by regulating TGF-β1/Smad3 and NF-κB pathway. Meanwhile, sodium tanshinone IIA sulfonate 33 can improve the bladder fibrosis in rats with partial bladder outlet obstruction by inhibiting the activation of TGF-β/Smad pathway.

There are many microRNAs have been described involved in tendon injuries such as miR-210 which could improve tendon injury healing via regulation of angiogenesis and miR-29b that could prevent tendon adhesion. Also, the miR-29b has been shown to play an essential role in different diseases such as diabetes, cancer, and heart 34-36. In particular, our previous study showed that TSA treatment prevented tendon adhesion through TGF-β/Smad3 pathway and at the same time could upregulate the expression of miR-29b. Of note, studies have been described that miR-29b is highly enriched in the nucleus compared with the cytoplasm. However, the mechanisms are not very clear 37. One of the possible reasons is that miR-29b could modulate the transcript factors or key genes involving in TGF-β/Smad3 pathway.

Considering that TSA and mir-29b both can regulate tissue fibrosis through TGF-β/Smad3 pathway, therefore in our study, we combined these two kinds of treatment, successfully established an adenoviral vector system to transfer miR-29b shRNA to primary cells and injected TSA afterwards. Animal models and our model showed that the combination of two treatments not only prevent tendon adhesion but also enhance the strength of the tendon, for the use of mir-29b inhibitor at the broken end of tendon can start the endogenous repair mechanism, and then the use of TSA can inhibit the exogenous repair mechanism, especially after the treatment of mir-29b inhibitor for 6 hours in our rat model, TSA can produce less cytotoxicity, and the effect of inhibiting tendon adhesion and enhancing the strength of tendon adhesion after healing is better. However, there are still some remaining questions need to be further addressed such as the way to turn on/off the expression of miR-29b after tendon healing, it will be very meaningful to bring this concept to clinical practices.

## Conclusion

In this study, we performed both *in vitro* and *in vivo* experiments to assess the effects of the combination of TSA and miR-29b inhibitor on tendon adhesion and tendon strength. Our data demonstrated that the use of miR-29b inhibitor at the end of tendon break could initiate endogenous repair mechanism and subsequently use of TSA should be able to inhibitor exogenous repair mechanisms. Therefore, our study highlights the possibility that the combination of both treatments could prevent tendon adhesion and ensure tendon strength.

## Supplementary Material

Supplementary figures and table.

## Abbreviations

TSA: Tanshinone IIA
miR-29: miRNA-29
H&E: hematoxylin and eosin staining
FACS: fluorescence-activated cell sorting
TAA: thioacetamide
