# Supplementary material for: Improved tendon healing by a combination of Tanshinone IIA and miR-29b inhibitor treatment through preventing tendon adhesion and enhancing tendon strength
Source: Int J Med Sci. 2020 Apr 27;17(8):1083–94. doi: 10.7150/ijms.44138 (PMC7211157; doi:10.7150/ijms.44138)
Supplement: Supplementary file 1 — Supplementary figures and table. [file ijmsv17p1083s1.pdf]

Figure S1: A: a representative figure of Western blotting. B: cell viability was checked by Annexin V assay by flow cytometry. C: cell cycle was detected by staining followed with flow cytometry analysis.

Figure S1

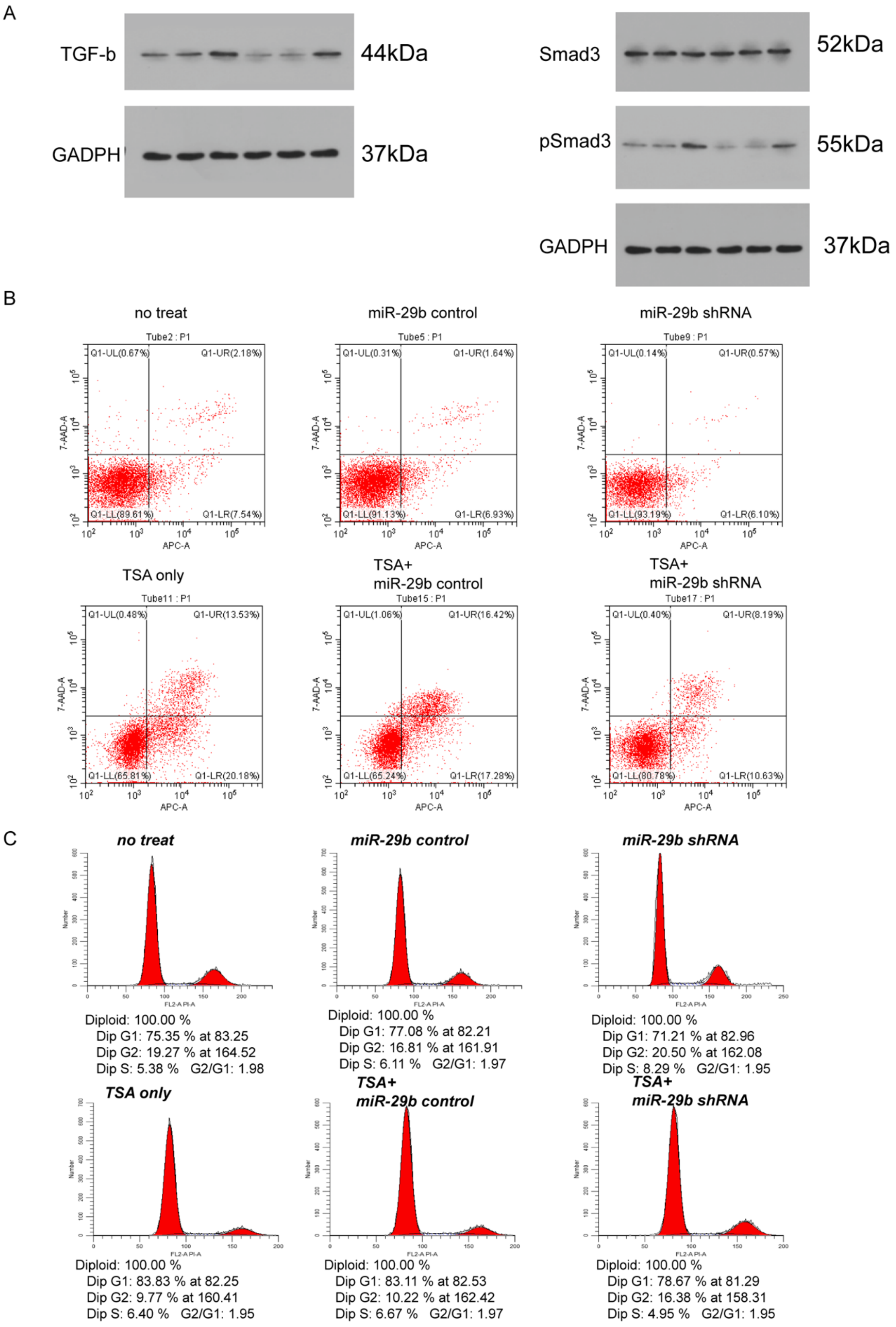

Figure S2: TUNEL assay was performed to look at the frequency of apoptotic analysis. The apoptotic cells were labelled in red and nucleus was in blue.

Figure S2

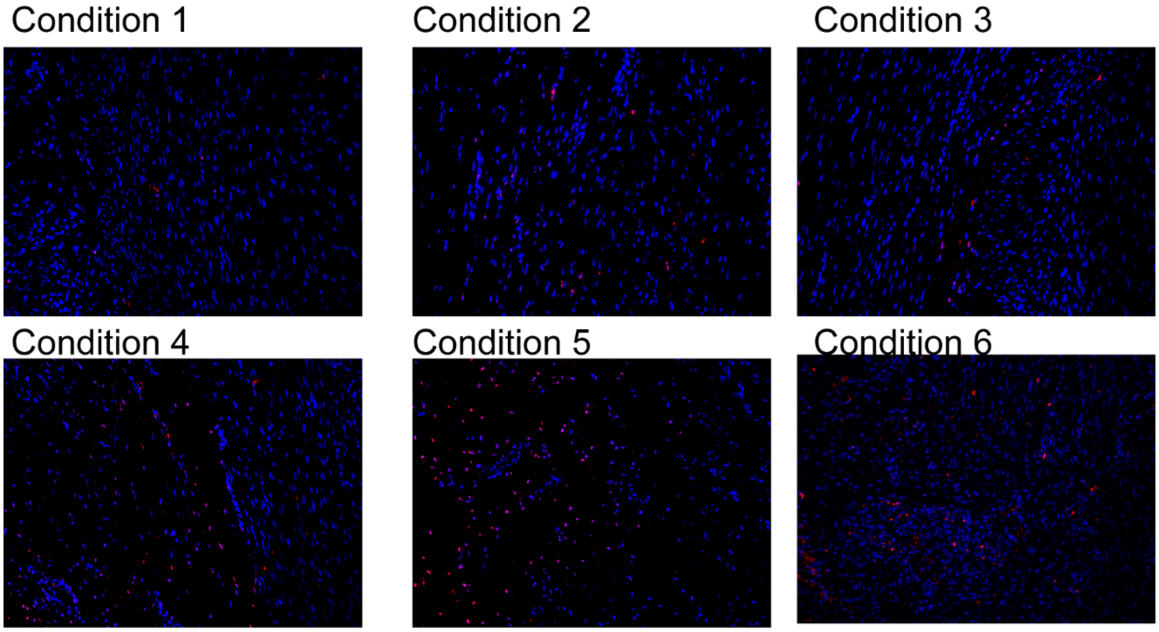

1. control group
2. model group+ no treat
3. model group +miR-29b inhibitor+TSA II(0h)
4. model group +miR-29b inhibitor+TSA II(6h)
5. model group +miR-29b inhibitor+TSA II(24h)
6. model group +miR-29b inhibitor+TSA II(72h)

Figure S3: Analysis of tendon tissue on collagen I, III, and cyclin D protein expression under six different conditions.

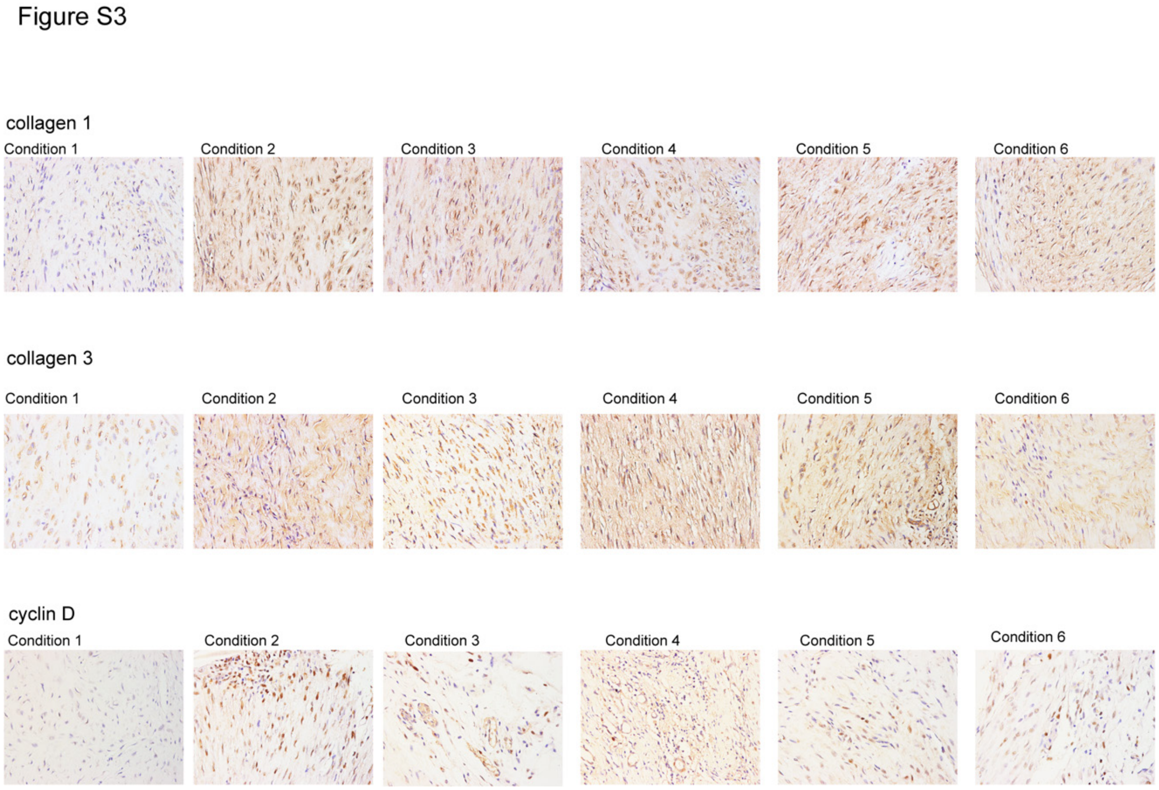

Table S1: primes used in this study

Primers in this study

Name	Primer	Sequence
U6	Forward	5‘- CGCTTCGGCAGCACATATAC -3’
	Reverse	5‘- AAATATGGAACGCTTCACGA -3’
rno-miR-29b	Forward	5‘- TGCGC TAGCACCATTTGAAATCA-3’
	loop primer	5‘- GTCGTATCCAGTGCAGGGTCCGAGG TATTCGCACTGGATACGACAACACT GA -3’

Rat GAPDH	Forward	5‘- ACAGCAACAGGGTGGTGGAC -3’
	Reverse	5‘- TTTGAGGGTGCAGCGAACTT -3’
Rat TGF-β1	Forward	5’- CACTCCCGTGGCTTCTAGTG-3’
	Reverse	5’- GGACTGGCGAGCCTTAGTTT-3’
Rat P21	Forward	5’-ACAGCAGGTCAAGAGGAGTA-3’
	Reverse	5’-CTGAGCCTGTTTCGTGTCTA-3’
Rat Smad3	Forward	5’-CCTCCAATTCAGAGCGCTTC-3’
	Reverse	5’-ATAGCACTGTCACTGAGGCA-3’
